# Cellular cross-talk drives mesenchymal transdifferentiation in diabetic kidney disease

**DOI:** 10.3389/fmed.2024.1499473

**Published:** 2025-01-07

**Authors:** Arunita Chatterjee, Jacqueline Tumarin, Sharma Prabhakar

**Affiliations:** Department of Internal Medicine, Texas Tech University Health Sciences Centre, Lubbock, TX, United States

**Keywords:** diabetes mellitus, kidney disease, cellular cross-talk, EMT, endothelial-to-mesenchymal transition, SGLT-2 inhibitors, myofibroblast transdifferentiation

## Abstract

While changes in glomerular function and structure may herald diabetic kidney disease (DKD), many studies have underscored the significance of tubule-interstitial changes in the progression of DKD. Indeed, tubule-interstitial fibrosis may be the most important determinant of progression of DKD as in many forms of chronic glomerulopathies. The mechanisms underlying the effects of tubular changes on glomerular function in DKD have intrigued many investigators, and therefore, the signaling mechanisms underlying the cross-talk between tubular cells and glomerular cells have been the focus of investigation in many recent studies. Additionally, the observations of slowing of glomerular filtration rate (GFR) decline and reduction of proteinuria by recent drugs such as SGLT-2 blockers, whose primary mechanism of action is on proximal tubules, further strengthen the concept of cross-talk between the tubular and glomerular cells. Recently, the focus of research on the pathogenesis of DKD has primarily centered around exploring the cross-talk between various signaling pathways in the diabetic kidney as well as cross-talk between tubular and glomerular endothelial cells and podocytes with special relevance to epithelial-to-mesenchymal transition (EMT) and endothelial-to-mesenchymal transition (EndoMT). The focus of this review is to provide a general description of cell-to-cell cross-talk in the diabetic kidney and to highlight these concepts with evidence in relation to the physiology and pathophysiology of DKD.

## Introduction

The incidence and prevalence of diabetes mellitus (DM) have reached epidemic proportions globally, with recent reports suggesting that as many as 600 million people are affected globally, and it is on the rise. By 2030, the prevalence of DM is expected to increase by >50% (1). Furthermore, the International Federation of Diabetes estimates that the prevalence will reach over 0.7 billion people in the world by 2045 (2). With the increase in diabetes, there will be an expected increase in the prevalence of vascular complications of diabetes, particularly diabetic kidney disease (DKD). The natural course of DKD includes, in most cases, the onset and progression of proteinuria, the development of increasing hypertension, and the incremental decline of glomerular filtration rate (3). The most important pathogenic factors contributing to the genesis and progression of DKD stem from both metabolic and hemodynamic derangements. Activation of the renin-angiotensin-aldosterone system (RAAS), often initiated by persistent uncontrolled hyperglycemia, leads to intraglomerular hypertension and hyperfiltration, resulting in proteinuria. A series of pathophysiologic events that occur sequentially and some concurrently result in epithelial-to-mesenchymal transition and fibrosis of nephronal units and renal parenchyma, resulting in end-stage renal disease (4). Intertwined in these pathogenic events is a constant dialogue amongst the various cells of the glomeruli and other components of the nephron—a cellular cross-talk that is instrumental in the continuous renal structural and functional decline—which is the focus of this manuscript.

## Pathophysiology of DKD—an overview

The kidney is one of the most common organs affected by long-standing diabetes, especially when uncontrolled, being seen in about 30–40% of diabetes patients (5). However, with an expanding spectrum of renal manifestations, including the non-proteinuric forms, the prevalence may increase to almost 50% of all diabetic subjects. The earliest histological changes in the diabetic kidney include mesangial expansion (including hypercellularity and increased matrix production) and thickening of the glomerular basement membrane (3). While these changes may be a result of metabolic derangements, the early functional changes in the kidney, such as glomerular hyperfiltration and microalbuminuria, are direct consequences of hemodynamic alterations caused by activation of RAAS and dysregulated nitric oxide (NO) metabolism. There is an initial increase in renal NO production driven primarily by endothelial NO synthase (eNOS) as an effect of sheer stress in the endothelium, which accounts for glomerular afferent vasodilation (6). Together with efferent arteriolar vasoconstriction mediated by angiotensin II as a part of RAAS activation, these intrarenal hemodynamic alterations lead to glomerular hypertension and hyperfiltration, leading to the onset of microalbuminuria. With continued hyperglycemia, there is activation of cellular cytokines such as protein kinase C and later transforming growth factor beta (TGF-β), which together initiate glomerular and tubule interstitial fibrosis. Other growth factors such as connective tissue growth factor (CTGF), platelet-derived growth factor (PDGF), and vascular endothelial growth factor (VEGF) also play a part in the onset and progression of renal fibrosis. Furthermore, continued uncontrolled hyperglycemia in the long term leads to a significant increase in the glycosylation of various proteins and accumulation of their break-down products or advanced glycosylation end products (AGE) (7). There are several deleterious consequences of AGE accumulation, including oxidative stress, NO quenching contributing to NO deficiency, and the formation of peroxynitrite, which has major cytotoxic effects (8). In addition, most of the AGE compounds, such as carboxymethyl lysine, acting through the receptors for AGE, trigger a series of intracellular signals that have significant vascular and cellular toxic effects. Furthermore, several pathogenic stimuli activate genes such as N-cadherin, Vimentin, TGF-β, and activated Wnt/β-catenin pathway that results in epithelial-to-mesenchymal transition (EMT) that leads to the transformation of renal epithelial cells in the glomeruli and tubules into myofibroblasts which ultimately leads to glomerular and tubule interstitial fibrosis (9). Similar activation of genes promoting endothelial-to-mesenchymal transition (EndoMT) results in the transformation of glomerular renal vascular endothelial cells into myofibroblasts, contributing to renal fibrosis (10).

Systemic hypertension is an important component of the clinical presentation of DKD. Hypertension is pure of renal origin in the nephropathy of type 1 diabetes, while it is an integral part of the metabolic syndrome in the nephropathy of type II diabetes (11). As the disease progresses, the prevalence and severity of hypertension increase so that when end-stage renal disease is reached, the hypertension is severe and universal. The presence of arteriolosclerosis is an essential part of renal histology in DKD (4).

Tubulointerstitial cellular infiltration and edema culminating in fibrosis is also a consistent feature in renal histology of DKD, although the severity of such changes is variable. It is widely established that interstitial fibrosis and tubular atrophy (IFTA) are frequent components and important contributors to renal functional decline in DKD. In addition, certain tubular functional derangements in DKD may influence glomerular hemodynamics and glomerular filtration rate (GFR) through tubule-glomerular feedback. Furthermore, manipulating certain transport processes in the tubules may impact glomerular pathology in DKD, as exemplified by how sodium-glucose cotransporter 2 (SGLT-2) blockers, which inhibit tubular reabsorption of glucose and sodium in the proximal tubule, improve GFR decline in DKD (12).

Intraglomerular and intra-nephronal cellular cross-talk: With such a complex interplay of pathogenic pathways and signaling cascades, an intricate and often bidirectional cross-talk can be envisioned to occur within the kidney in the evolution and progression of DKD. In the past decade, this aspect of DKD has been the focus of investigation, and evidence for such cell-to-cell cross-talk in the kidney is emerging and accumulating in the scientific literature. It is evident that such cross-talk occurs between different cells in the glomeruli (e.g., endothelial cells and podocytes, mesangial cells and podocytes) but also between different nephronal segments such as tubular epithelial cells and podocytes. A detailed discussion of such cell-to-cell dialogue and cross-talk involved in the pathogenesis is the primary content of this review.

## Cellular cross-talk in the glomerulus

In the kidney, the glomerulus is the complex filtering apparatus that has several cellular and non-cellular structural components that contribute to the glomerular filtration process or barrier. These include the glomerular basement membrane (GBM), glomerular endothelial cells (GECs), podocytes (PCs) with their foot processes, and the slit diaphragm. Mesangial cells (MCs) are located in close proximity to GECs and the GBM, often with cellular protrusions extending into the capillary level in between the GECs, providing direct contact for a potential cross-talk with GECs (13). MCs play a role in modulating the filtering surface area of the glomerulus by their contractile effects on GECs, an effect mediated by angiotensin II, thereby regulating the GFR (14). Angiotensin II also plays a role in mesangial expansion, which ultimately leads to glomerulosclerosis. Mesangial expansion has been linked to podocyte loss, underscoring the potential MC-PC cross-talk. GECs have a fenestrated surface, and the apical side is negatively charged, contributing to the filtrating function of the glomerulus. In the context of DKD, the hyperglycemia-mediated effects such as oxidative stress (generation of reactive oxygen species (ROS)) and nitrosative stress (decreased eNOS mediated NO production) along with pro-inflammatory effects lead to endothelial injury, dysfunction, and apoptosis resulting in albuminuria (8, 15). Furthermore, hyperglycemia promotes endothelial-to-mesenchymal transition (EndoMT) through activation of TGF-β whereby GECs acquire mesenchymal cell characteristics, leading to glomerulosclerosis (16). Podocytes (PCs) are located on the outer side of the GBM, with foot processes touching the GBM, and the contraction of the latter also regulates the glomerular filtration barrier. Hyperglycemia causes structural changes in PC, including foot process effacement, EMT, and apoptosis in DKD. Wnt-β catenin and phosphoinositide 3-kinase (PI3K)/Akt signaling pathways triggered by hyperglycemia may promote EMT in PCs. These changes in PCs lead to podocyturia and result in albuminuria and, ultimately, glomerulosclerosis. The cross-talk that takes place between the various cells in the glomerulus, such as GECs and PCs, PCs and MCs, GECs and MCs, can be broadly discussed under two stages of kidney disease in diabetes, namely early DKD and late DKD.

### Cellular cross-talk in early DKD

In the early stages of DKD, there is a loss of podocytes and mesangial cells with podocyturia. Secretion of Semaphorin 3C (SEMA3C) from MCs initiated by hyperglycemic conditions causes endocytosis of microtubules and increases the glomerular permeability by altering GEC permeability characteristics through the actions of Neuropilin-1 (NRP1) and Neuropilin-2 (NRP2) (17). These processes in early DKD exemplify one of the many instances of cell-to-cell cross-talk that occurs between MCs and GECs and initiates early renal damage. Persistent hyperglycemia in DKD activates NADPH oxidases in GECs, which leads to increased ROS, which triggers PC activation and mesangial expansion and contributes to proteinuria and progression of DKD. Activation of transforming growth factor beta (TGF-β) in GECs may play a role in mesangial expansion that ultimately leads to mesangial sclerosis. With continued expansion, mesangium expands into GECs, decreasing the filtration area and GFR. This constitutes a major structural change in DKD with important functional implications. Exosomes from high glucose (HG)-treated GECs upregulate the expression of genes for Fibronectin and Collagen IV in MCs that contribute to mesangial expansion (18). Another factor that contributes to mesangial expansion is the activation of the mechanistic target of rapamycin complex (mTORC) in PCs (19). Thus, a cross-talk involving all three major glomerular cells (GECs, PCs, MCs) is involved in mesangial expansion. In cell culture studies, GECs exposed to HG upregulated Endothelin 1 (ET-1) secretion, which binds to the Endothelin A receptor on MCs and promotes MC proliferation mediated by the RhoA-Rho kinase (ROCK) pathway (20). Furthermore, downregulation of genes related to the extracellular matrix (ECM) was observed in podocytes co-cultured under HG conditions along with GECs but not when PCs were cultured alone, underscoring the role of GEC-PC cross-talk in mediating this effect (21). Recent studies support the upregulations genes Early growth response-1 (EGR-1) as well as EGR-2 and EGR-3 in GECs and PCs under HG conditions, genes that mediate expression of mesangial matrix proteins, supporting the GEC-PC-MC cross-talk in early DKD (22).

### Cellular cross-talk in the late phase of DKD

**Figure 1:**
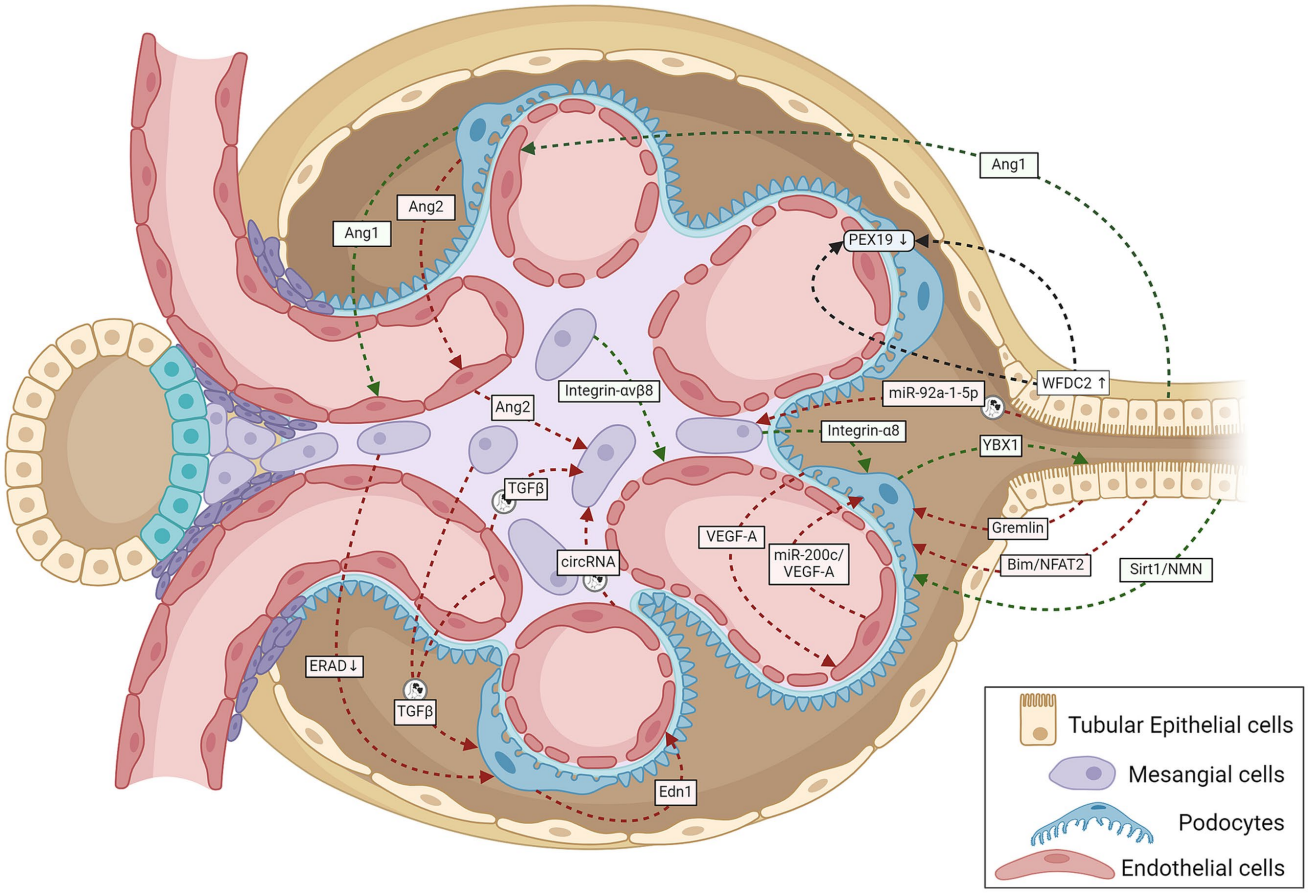
A schematic representation summarizing cell-to-cell cross-talk amongst GECs, MCs, podocytes, and TECs within the renal corpuscle relevant to the onset and progression of DKD. Green lines indicate protective signaling; red lines, pathogenic; and black lines, unknown whether pathogenic or protective. The circular exosome icons show communications known to be mediated via exosomes containing proteins or RNA. Angiogenesis disorders and podocyte injury mark the initial stage of DKD. Normal podocytes abundantly express and secrete VEGF-A and Ang1, and injury reduces their expression; both proteins are necessary for GEC survival and growth (107, 108). Tubular cells also produce the GEC-protective Ang1 (46). Stressed GECs produce miR-200c, which impairs glomerular homeostasis by targeting podocyte-produced VEGF-A (68). Podocyte-specific over-expression of Ang2 causes GEC apoptosis (109). The diabetic milieu causes GECs to release Ang2, which also induces MC apoptosis (110). Glucose-stressed MCs can inhibit physiological ERAD, suppress nephrin phosphorylation, and induce apoptosis in podocytes, escalating podocyte injury (28). Podocyte-produced Edn1 promotes GEC dysfunction, and SENP6 represses Edn1 expression in podocytes, thereby protecting GECs from injury (111). Podocytes produce the cold shock protein Y-box–binding protein 1, which has been implicated in various kidney diseases. Rana et al. show that YBX1 secreted by podocytes inhibits TLR4–NF-κB signaling and, thereby, sterile inflammation in tubular epithelial cells (112). With podocyte injury, the absence of these protective cues would leave the tubular epithelial cells more vulnerable to the diabetic inflammatory environment, as suggested by mutant mice (deletion in podocytes or secretion-deficient YBX1) with disrupted YBX1-dependent glomerular-tubular interaction being predisposed to LPS-induced kidney injury (112). Mesangial expansion and sclerosis with ECM production characterize the intermediate stages of DKD, with the changing ECM protein profile potentially altering the inter-cellular cross-talk. Expressed specifically in MCs, integrin-α8 protects glomerular integrity, esp. during hypertension and diabetes. Lack of integrin-α8 aggravates podocyte injury in experimental DKD (113). Similarly, MC expressed integrin-αvβ8 protects GECs by sequestering TGF-β in its latent conformation (38). Cells under stressful conditions release more exosomes with different communication signals: differences in levels and types of proteins and coding and regulatory RNAs. Glucose-stressed GECs secrete exosomes enriched in TGF-β1 mRNA and circRNA, which activate MCs promoting renal fibrosis (18, 37) and induce EMT and dysfunction in podocytes (34). Similarly, MCs stressed with high glucose levels also secrete TGF-β1–enriched exosomes, which increase podocyte apoptosis and reduce their adhesion (29). Proximal TEC-derived exosomal miR-92a-1-5p induces ER stress and myofibroblast transdifferentiation of MCs, resulting in DKD progression (44). Other known communication between TECs and glomerular cells include (i) TEC-produced Sirt1 suppressing Claudin-1 overexpression in podocytes (41), (ii) Gremlin overexpression in TECs worsening diabetes-induced glomerular damage (39), (iii) TEC-produced Bim inducing podocyte cytoskeletal dysfunction via NFAT2 and a lncRNA (40), and (iv) TEC-secreted WFDC2 downregulating PEX19 in podocytes and GECs in DKD (114). Ang1, angiopoietin 1; Ang2, angiopoietin 2; Bim, B cell lymphoma-2 interacting mediator of cell death; circRNA, circular RNA; Edn1, endothelin 1; ERAD, endoplasmic reticulum-associated degradation; GECs, glomerular endothelial cells; MCs, mesangial cells; NFAT2, nuclear factor of activated T cells 2; NMN, nicotinamide mononucleotide; PEX19, peroxisomal biogenesis factor 19; SENP6, small ubiquitin-like modifier (SUMO) specific peptidase 6; Sirt1, Sirtuin 1; TECs, tubular epithelial cells; TGF-β1, transforming growth factor-β1; VEGF, vascular endothelial growth factor; WFDC2, whey acidic protein four-disulfide core domain protein 2; also known as human epididymis protein 4 (HE4); YBX1, Y-box–binding protein 1.

In the later phases of DKD, structural alterations culminate in glomerular and tubulointerstitial fibrosis. Several functional disturbances in the glomeruli that occur in the late DKD and result in renal fibrosis are mediated by an intricate and continuous cross-talk between all the major cells in the kidney. HG-treated GECs secrete exosomes that deliver circular RNA (circRNA) and microRNA (miRNA) to MC, which then produce ECM proteins and initiate renal fibrosis. Loss of glucocorticoid receptor (GR) in the podocytes leads to abnormal activation of the Wnt signaling cascade and affects the integrity and function of GECs, leading to functional decline in late DKD (23). Using ligand analysis, recent studies confirmed closer interactions between fibroblasts with all major cell types in the nephrons from DKD subjects compared to diabetic subjects with no kidney disease or non-diabetic subjects, an observation linked to the interplay of chemokines from fibroblasts with various renal cells (24). These findings establish the close cell-to-cell interactions between various renal cells and renal fibrosis. Figure 1 summarizes the major communication amongst the major renal cell types.

The following section describes specific examples of cross-talk between different glomerular cells in DKD.

### Cross-talk between podocytes and mesangial cells

Mesangial cells participate in glomerular cell-to-cell cross-talk through vascular endothelial growth factor (VEGF) signaling pathways. Overexpression of VEGF-A in PCs has been shown to affect MC markers, such as desmin, α-SMA, PDGFR-β, and mesangial expansion (25). Furthermore, VEGF-A from PCs has been shown to affect MC differentiation and proliferation (26). Another significant cross-talk that occurs between PCs and MCs involves ET-1-mediated effects. Experimental studies indicate that inhibition of ET-1 receptors or deletion of ET-1 receptors A and B in PCs inhibits MC proliferation and mesangial expansion through inhibition of Wnt-β catenin and NF-κB signaling mediated by ET-1 receptors (27).

Endoplasmic reticulum stress (ERS) has been shown to contribute to the development and progression of DKD. In vitro studies have shown that elutes from MC cultures in HG inhibited ER-associated degradation (ERAD)-related proteins such as Derlin-1 and Derlin-2 in PC, leading to ERS, which amplified podocyte loss, albuminuria, and DKD progression (28).

Additional evidence of PC-MC cross-talk comes from the cell culture studies where HG-exposed MCs were shown to secrete copious exosomes containing TGF-β1, which upregulate TGF-β1 receptor expression on PC. These effects promote PC apoptosis through the TGF-β1-PI3K/Akt signaling pathway (29).

### Cross-talk between podocytes and glomerular endothelial cells

Vascular endothelial growth factor A (VEGF-A), an angiogenic factor produced by podocytes, affects the proliferation and permeability of glomerular capillaries acting through VEGF receptors (VEGFR) 1 and 2 expressed on the surface of GECs. Initially, there is an increased production of VEGF from PCs and increased expression of VEGFR-1 and-2 in GECs in early DKD. These changes result in increased production of NO in the glomerulus through increased eNOS expression and contribute to hyperfiltration and microalbuminuria (30). The increased VEGF-A in early DKD from PCs acting on GECs promotes neo-angiogenesis, which delays fibrogenic processes in the initial stages of DKD. However, as the disease advances, there is a decline in both VEGF-A from PCs and VEGFR-1 and-2 expression in GECs, leading to decreased glomerular eNOS and NO levels contributing to renal fibrosis (6).

Activation of the TGF-β1 receptor on PCs leads to the synthesis of Endothelin-1 (ET-1) from pre-ET-1 through the Smad signaling pathway and acting through ET-1 receptor A located on GECs results in mitochondrial oxidative stress in GECs (31). This illustrates another example of PC-GEC cross-talk leading to podocyte-mediated endothelial cell injury in DKD.

Mitochondrial oxidative stress has also been incriminated in the progression of DKD. Experimental studies examining the effects of HG on GECs demonstrated evidence of significant mitochondrial oxidative stress with increased production of mitochondrial superoxide and 8 hydroxy deoxyguanosine (8 OHdG) and decreased eNOS activity with consequent endothelial dysfunction. Supernatants from such culture studies, when transferred to PCs in culture, promoted PC apoptosis, an effect that was abolished by the elimination of mitochondrial superoxide by TEMPO (32). These observations imply a significant interaction between GECs and PCs, mediated by mitochondrial oxidative stress.

Another instance of PC-GEC cross-talk is illustrated by the effects of NO derived from GECs through the action of eNOS, which affects the structural and functional integrity of PCs since eNOS deficiency results in PC apoptosis (33). Exosomes secreted from HG-treated GECs containing TGF-β mRNA are endocytosed by PCs, which triggers EMT through activation of the Wnt-β catenin signaling pathway (34). There is additional evidence from animal studies about GEC-PC cross-talk involving TGF-β signaling in DKD mediated by bone morphogenic protein-activin membrane-bound inhibitor (BAMBI). BAMBI negatively regulates TGF-β signaling in both GECs and PCs and, therefore, in GEC-BAMBI−/− diabetic mice, podocyte injury and loss were observed, indicating a complex interplay between these cells mediated by TGF-β/Smad signaling pathways (35).

### Cross-talk between mesangial cells and glomerular endothelial cells

Nitric oxide (NO) has been shown to play a significant role in the onset and advancement of DKD. Nitric oxide is produced from L-arginine, which is the sole substrate for NO generation, a reaction catalyzed by nitric oxide synthases (NOS). All cells in the entire nephron are capable of producing NO, although in variable capacities. The NO production in GECs and podocytes and tubular epithelial cells (TECs) is generally mediated by constitutive NOS (cNOS or eNOS), while in MCs, the reaction is mediated by inducible NOS (iNOS), which requires inflammatory cytokines such as *λ*-Interferon or lipopolysaccharide or TNF-α to stimulate MCs (30). In the early phases of DKD, there is increased intrarenal NO primarily from iNOS mediated from MCs (with modest contribution from eNOS activation), which leads to afferent glomerular arteriolar vasodilation. Together with angiotensin-mediated efferent arteriolar constriction, the intraglomerular pressure increases, leading to hyperfiltration and microalbuminuria. With progressive DKD, the mitochondrial oxidative stress with increased ROS generation and NO quenching by AGE decreases NO availability, leading to endothelial dysfunction in GECs.

In vitro studies also supported a fine cross-talk between GECs and MCs through NO-dependent mechanisms. In co-cultures involving GECs and MCs, incubation with bradykinin increased intracellular cyclic-GMP (cGMP) in MCs in a NO-dependent pathway. As bradykinin stimulates eNOS present in GECs, the NO released as a result of such eNOS activation leads to cGMP generation in MCs (36). These observations confirm the NO-mediated cross-talk between GECs and MCs.

An important example of GEC-MC cross-talk involves the TGF-β signaling pathway. HG-treated GECs secrete exosomes containing TGF-β mRNA, which are then endocytosed by MCs and, through Smad signaling, result in MC proliferation, mesangial expansion, and secretion of ECM proteins (18). Exosomes containing circular RNA from HG-treated GECs, when taken up by MCs, induced the expression of proteins that caused mesenchymal transdifferentiation (37). Another evidence of GEC-MC cross-talk also involves TGF-β signaling. Inhibition of MC expression of integrin αvβ8 in experimental diabetes resulted in decreased binding of latent TGF-β and releasing excess TGF-β to reach GECs, resulting in GEC apoptosis (38).

## Cross-talk between tubular epithelial cells and glomerular cells

As discussed previously, although glomerular pathology is well known and described in DKD, the majority of patients with proteinuria in diabetes have significant pathology. In addition, tubular and interstitial changes, such as cellular infiltration and edema, may not only precede glomerular changes but may have a greater impact on the progression of DKD. With the progression of DKD, the tubules undergo atrophy and scarring along with interstitial fibrosis. Thus, the structural and functional changes in the tubulo-interstitium have a significant impact on the glomerular structure and function in DKD. It is, therefore, conceivable that there could be major interactions between the TECs and various glomerular cells. A discussion of various established and evolving data pertaining to the cross-talk between these cells is presented in the following paragraphs of this section and summarized in Table 1.

**Table 1:** Tubulo-glomerular cross-talk.

Cell-types involved	Mediators	Mechanisms/Pathway	Effect on DKD	Reference
TEC→GEC	Ang1	Diabetes-associated changes in expression of Ang1 in distal TECs and collecting ducts Ang1, a secreted glycoprotein, functions by binding its receptor Tie-2 expressed on endothelial cells	Ang1 protects vasculature, suppresses plasma leakage, inhibits vascular inflammation, and prevents endothelial death, and a reduction of Ang-1 expression in DKD reduces the protective effects	(46)
TEC→GEC, podocyte	WFDC2	Downregulates PEX19 in podocytes and GECs	Unknown	(114)
TEC→MC	Exosomes with miR-92a-1-5p	TEC-secreted exosomes containing miR-92a-1-5p promote ER stress and myofibroblast transdifferentiation in MCs	Contributes to renal fibrosis High urinary levels of miR-92a-1-5p correlate with low eGFR levels	(44)
TEC→MC, podocyte	Gremlin	Antagonizes BMPs Increases TGF-β Increases inflammation	Causes significant mesangial expansion, GBM thickening, podocytopenia	(39)
TEC→podocyte	Bim	Activates NFAT2 in TECs and downregulates lncRNA NONHSAT179542.1 in podocytes	Contributes to podocyte cytoskeletal damage.	(40)
TEC→PEC, podocyte	Sirtuin 1	Anti-inflammatory Anti-fibrotic Anti-apoptotic Suppresses Claudin-1 overexpression in PECs, podocytes Maintains NMN concentrations around glomeruli	The protective influence of PTEC-Sirt1 on podocytes diminishes in DKD, allowing podocyte effacement and proteinuria	(41)
Podocyte→TEC	YBX1	Podocyte-secreted cold shock protein YBX1 reduces tubular inflammation by binding to and inhibiting TLR4 signaling	Podocyte injury in DKD removes the podocyte-to-TEC protection, thus leaving the tubular cells susceptible to inflammation and injury	(112)

This table summarizes known communication between renal TECs and glomerular cells. Ang1, angiopoietin 1; BMP, bone morphogenetic protein; eGFR, estimated glomerular filtration rate; ER, endoplasmic reticulum; GEC, glomerular endothelial cell; GBM, glomerular basement membrane; lncRNA, long non-coding RNA; MC, mesangial cell; NFAT2, nuclear factor of activated T cells 2; NMN, nicotinamide mononucleotide; PEC, parietal epithelial cell; PEX19, peroxisomal biogenesis factor 19; TEC, tubular epithelial cell; TGF-β, transforming growth factor beta; TLR4, toll-like receptor 4; WFDC2, whey acidic protein four-disulfide core domain protein 2, also known as human epididymis protein 4 (HE4), YBX1, Y-box–binding protein 1.

### Cross-talk between tubular epithelial cells and podocytes

There are several examples of PC-TEC cross-talk in DKD. A major instance of such interaction involves bone morphogenic proteins (BMPs) that belong to the TGF-β superfamily group. While TGF-β is a fibrogenic factor, BMPs counterbalance the TGF-β effect. Gremlin is an antagonist of BMP that is overexpressed in TECs, MCs, and PCs when exposed to HG. Animal studies have shown that in transgenic diabetic mice for overexpressing tubule-specific Gremlin, PC damage was exaggerated with severe foot process effacement and podocyte loss (39). These observations suggest tubular overexpression of Gremlin resulted in a cross-talk with PCs, leading to major podocytopathy.

Another example of cross-talk between TECs and PCs involves Bcl2 interacting mediator of cell death (Bim), an apoptotic mediator. Culturing proximal TECs (PTECs) in HG increased Bim expression. Xu et al. showed that when PCs and PTECs were co-cultured, increased Bim expression in PTECs resulted in significant damage to PCs due to increased synaptophysin and F-actin expression. Furthermore, if the PTECs were transfected with Lenti-Bim shRNA and treated with HG, the changes in cocultured in PCs described earlier disappeared, confirming that the cross-talk between PTECs and PCs is mediated by Bim (40).

Hasegawa et al. showed in streptozotocin (STZ)-treated transgenic mice expressing proximal tubule-specific Sirtuin 1 (Sirt1, a nicotinamide adenine dinucleotide-dependent deacetylase, which counters the effects of NF-κB, TGF-β, and p53 signaling) reduced albuminuria and showed better renoprotection compared with control STZ mice (41). Furthermore, such transgenic mice also demonstrated decreased Claudin-1 expression in PCs and parietal epithelial cells of glomeruli, minimizing the epithelial cell damage. Nicotinamide mononucleotide (NMN) upregulated Sirt1 expression in TECs (42) and diminished the Claudin-1-mediated effects on PC damage, thereby reducing albuminuria. The authors concluded that these observations support the interaction of TECs and PCs in the regulation of Claudin-1 expression and effects in PCs mediated by Sirt1.

### Cross-talk between tubular epithelial cells and mesangial cells

There is limited literature investigating the cross-talk between TECs and MCs in the context of DKD. Previous experimental evidence supported the role of endothelial cell-myofibroblast transdifferentiation (MFT) in renal fibrosis (43). Recent studies involving co-cultures of PTECs (HK-2 Cells) with MCs in HG media have shown that exosomes from TECs expressing miR-92a-1-5p promoted MFT in MCs (44). The role of miR-92a-1-5p in this cross-talk and the role in DKD was confirmed by the observation of high levels of this exosomal miRNA in the urine of patients with advanced DKD.

### Cross-talk between tubular epithelial cells and glomerular endothelial cells

A major mediator of cross-talk between TECs and GECs is the Angiopoietin/Tie signaling system (45). Angiopoietin 1 (Ang 1) is predominantly distributed in the TECs and has salutary effects on the renal endothelial cell integrity and function, while Angiopoietin 2 (Ang 2) and Tie (a co-receptor for Ang 1 and 2) are expressed in the GECs and have detrimental effects on the function and integrity of capillaries. Immunohistochemical studies have suggested that as the DKD progresses, Ang 1 expression decreases (after an initial increase) steadily while Ang 2 increases. Thus, the endothelial preservation by the interaction of Ang 1 from TECs on GECs decreases while increasing the Ang 2/Ang 1 ratio promotes progressive endothelial injury with advancing DKD (46).

## SGLT2 inhibition and renal function preservation: implications for renal cellular cross-talk

**Figure 2:**
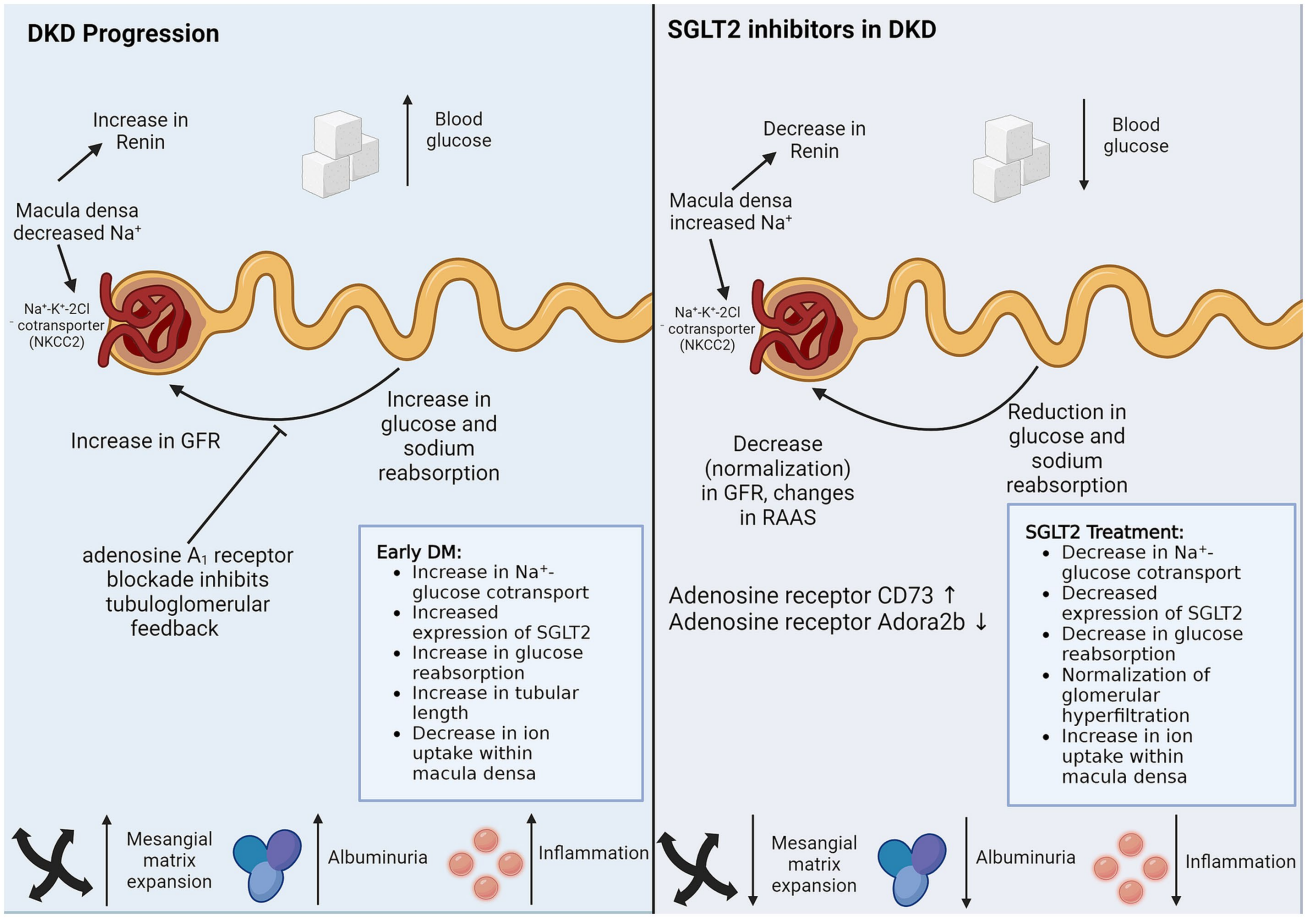
SGLT2 inhibitors in DKD. SGLT2 inhibitors reduce sodium and glucose reuptake by proximal TECs, thereby managing DKD via the two biggest threats, hypertension and hyperglycemia. SGLT2 inhibitors also have protective effects on cells that do not express SGLT2. Direct non-SGLT2 mediated effects of SGLT2 inhibitors and indirect cross-talk from SGLT2 expressing proximal TECs can explain the protective role of SGLT2 inhibitors on non-SGLT2-expressing cells. The figure illustrates the caveats of DKD progression, which are controlled by SGLT2 inhibitor treatment. DM, diabetes mellitus; DKD, diabetic kidney disease; GFR, glomerular filtration rate; RAAS, renin-angiotensin-aldosterone system; SGLT2, sodium-glucose cotransporter-2.

Recent major clinical trials have established that SGLT-2 inhibitors, a class of drugs that act by inhibiting the sodium-glucose cotransporter 2 (SGLT-2) located in the proximal tubule, are effective not only in lowering blood glucose levels in diabetic subjects but also cardio-protective and reno-protective. The reno-protective effects, in particular, have been very conclusive in studies such as DAPA-CKD (47) and Credence (48) that the new paradigms of treatment of DKD have advanced SGLT-2 blockers to the first line of therapy. While the basic mechanism of action of these agents for diabetic control is obvious, viz. by inhibiting glucose transport in the proximal tubule, thereby causing glucosuria and lowering plasma glucose (Figure 2), the basis of reno-protective effects of reducing the urine protein excretion rates and slowing the decline of GFR in DKD remains by far quite unclear.

The current understanding of the mechanisms of reno-protective effects of SGLT-2 blockers is based on three potential explanations. Firstly, the glucosuric effect of these agents is associated with significant natriuresis, which, through tubule-glomerular feedback regulation, results in afferent arteriolar vasoconstriction, thereby reducing hyperfiltration. Since glomerular hyperfiltration is a prime driver of albuminuria and a precursor of glomerulosclerosis, SGLT-2 blockers, by their effects on glomerular hemodynamics, would be reno-protective. Secondly, by decreasing the tubular reabsorption of glucose in the proximal tubule, the peritubular glucose concentration is reduced. Since glucose in the peritubular interstitial space promotes edema and tubulointerstitial fibrosis, SGLT-2 inhibitors would be expected to attenuate these effects. As interstitial fibrosis and tubular atrophy (IFTA) are strong predictors of renal functional decline, therapy with SGLT-2 blockers would result in reno-protection. Finally, the use of these agents would result in a ketotic state, and oxidation of ketones in preference to free fatty acids reduces the oxidative stress in target organs such as the heart and the kidney (49).

Despite the preceding discussion about the potential mechanisms of renoprotection by SGLT-2 blockade, most of this area remains scientific speculation, and several questions remain unanswered. One of the main questions is if cross-talk between the PTECs and glomerular cells, specifically GECs, is involved in mediating these effects. For example, it remains unknown if the natriuretic effects on afferent arterioles and glomerular hemodynamics involve a PTEC-GEC cross-talk that may be mediated by NO signaling. Similarly, the reno-protective effects of the SGLT-2 blockade may involve a cross-talk between TECs and PCs, an area that needs further investigation.

## Myofibroblast transdifferentiation in DKD

Myofibroblast transdifferentiation is an integral component of DKD pathogenesis. Accumulation of myofibroblasts initiates fibrosis. Renal interstitial myofibroblast accumulation correlates with ECM deposition and fibrosis severity. Myofibroblasts are the primary producers of the fibrotic ECM. They produce collagens, fibronectins, fibrillins, elastins, tenascins, and smaller molecules like proteoglycans and matricellular proteins like matrix metalloproteinases (MMPs) and tissue inhibitors of metalloproteinases (TIMPs) (50). Protein matrices aid in tissue degradation, blood flow inhibition, and structural stiffening. Myofibroblasts also release inflammatory cytokines and chemokines like IL-1α, IL-1β, IL-6, TNF-α, and CCL2, exacerbating interstitial damage (51–53).

Several cell types in the renal diabetic environment can transition into pathogenetic myofibroblasts, including resident fibroblasts, epithelial cells, endothelial cells, pericytes, and macrophages (53–55). Studies vary considerably in attributing the proportion of contribution from each cell type primarily because of differences in the cell-fate tracing methods used (53). Although researchers disagree on the proportion of the contribution of the MFT sources and are uncertain about MFT in humans, all studies agree that intercellular communication is at the heart of triggering MFT in any of the source cell types. Next, we discuss the role of intercellular cross-talk in driving MFT in the different implicated cell sources, acknowledging that several others might be evading researchers.

### Renal intercellular cross-talk in MFT of fibroblasts in DKD

Fibroblasts are proposed to be the primary sources of myofibroblasts instigating renal fibrosis in DKD. Fibroblast-to-myofibroblast transition (FMT) is the process of activated fibroblasts differentiating into myofibroblasts. Fibroblasts are stromal cells that help repair damaged tissues. They are activated by mechanical stress and paracrine signaling molecules.

In the context of DKD, renal cellular injury following the metabolic insults results in producers of factors required for promoting regeneration and tissue healing, such as TGF-β, platelet-derived growth factor (PDGF), hedgehog, and Wnt ligands. However, persistent injury causes sustained production of these ligands, resulting in paracrine fibrogenic effects (56).

The most prominent and best-studied fibroblast-activating signal is the cytokine TGF-β. Injured epithelia, macrophages, fibroblasts, endothelial cells, and mesangial cells are all potent sources of TGF-β. Active TGF-β1 binds its receptors and activates the canonical TGF-β/Smad signaling pathway in fibroblasts, resulting in the upregulation of its target genes, including α-SMA, ECM molecules, and integrin receptors. Meng et al. reviewed the various cell-type and context-dependent roles played by TGF-β in renal fibrosis (57). Wu et al. recently showed that macrophages undergoing MFT increase the expression of TGF-β, and the conditioned media from these cells activates fibroblasts-to-myofibroblast transition (58). Some integrin receptors, like integrins-αvβ5 and-αvβ6, participate in supplying more of the activated TGF-β. They bind to latent TGF-β, making their activating proteolytic cleavage sites available, thus releasing more of the activated TGF-β into the microenvironment (59–61). This results in a feed-forward loop whereby an activated fibroblast induces FMT in its vicinity. However, other integrin receptors, e.g., integrin-αvβ8 expressed by mesangial cells, have been suggested to protect their neighboring GECs by sequestering TGF-β in its latent form (38). Another connection between TGF-β and integrin signaling is via Yes-associated protein/transcriptional coactivator with PDZ-binding motif (YAP/TAZ). YAP and TAZ are transcription co-activators and, therefore, control gene expression. Stiff ECM promotes the nuclear localization of YAP/TAZ and where they interact with the activated Smad complex, resulting in α-SMA transcription (61).

PDGFs are another set of repair/fibrogenic molecules long known to be growth factors for fibroblasts and promoters of renal MFT. In particular, platelet-derived growth factor-BB (PDGF-BB) induces renal tubulointerstitial cell proliferation, myofibroblast formation, and tubulointerstitial fibrosis in a dose-dependent manner (62). Another growth factor signaling utilized by injured renal cells to activate fibroblasts is the Hedgehog pathway. Upon binding the secreted Hedgehog ligands, the membrane receptor Patched releases the protein smoothened (Smo), which facilitates the transcription of Gli targets. Reporter experiments in mice show that while the ligands are primarily expressed in tubular epithelial cells, the receptor Patches and targets of Gli are exclusively expressed in perivascular fibroblasts and pericytes, suggesting an epithelial-fibroblast paracrine cross-talk via hedgehog signaling (63).

Another group of intercellular communicators driving the MFT of fibroblasts are Wnts. Injury-induced tubular secretion of profibrotic factors has been suggested to be a major contributor to renal fibrosis following acute kidney injury. In assessing the identity of the factors and the necessity of tubular injury, Maarouf et al. found that Wnt1 produced by proximal tubules was sufficient to cause interstitial myofibroblast activation, proliferation, and increased ECM production without injury or, remarkably, any signs of inflammation (64). Constitutive activation of canonical Wnt/β-catenin signaling in murine interstitial pericytes and fibroblasts exhibits spontaneous myofibroblast differentiation even in the absence of injury. Activated myofibroblasts strongly express Wnt4 during renal fibrosis, suggesting autocrine activation in addition to paracrine signaling. However, Wnt4 from interstitial myofibroblasts is not necessary for MFT, probably because of the compensation of other Wnt ligands and Wnt4 from other cells, such as the collecting ductal cells (65).

In addition to the affected secreted factors, signaling pathways, and transcription factors, non-coding RNAs are being recognized to play regulatory roles in the changing renal microenvironment of DKD. In an article published earlier this year, Xing and Rodeo reviewed the emerging roles of non-coding RNAs, including microRNAs, long non-coding RNAs, and circular RNAs, in FMT and fibrotic diseases (66). These include both pro-fibrotic and antifibrotic regulatory RNAs, opening up new therapeutic possibilities. Not only do non-coding RNAs regulate the cellular environment, they are also directly responsible for intercellular cross-communication. For example, high-glucose stimulated proximal TECs secrete exosomes enriched with microRNA (miR)-92a-1-5p. Glomerular MCs take up these exosomes. miR-92a-1-5p then induces ER stress and myofibroblast transdifferentiation of these MCs, resulting in DKD progression (44). Similarly, glucose-stimulated GECs produce more exosomes enriched in circular RNAs, which activate MFT in glomerular MCs (37).

FMT is perhaps the primary contributor of myofibroblasts in fibrotic diseases, but the process is also vital to tissue repair and healing. Targeting this process for fibrosis prevention is going to need more research. For example, TGF-β inhibitors have not worked very well in the clinical setting yet, probably because of TGF-β’s pleiotropic nature and its importance in other systems like immunity, repair, and regeneration. Therefore, understanding the context-dependent differences in fibrosis versus repair/regeneration in the players and pathways involved in FMT is necessary before targeting this process for therapeutic interventions.

### Renal intercellular cross-talk in EMT in DKD

**Figure 3:**
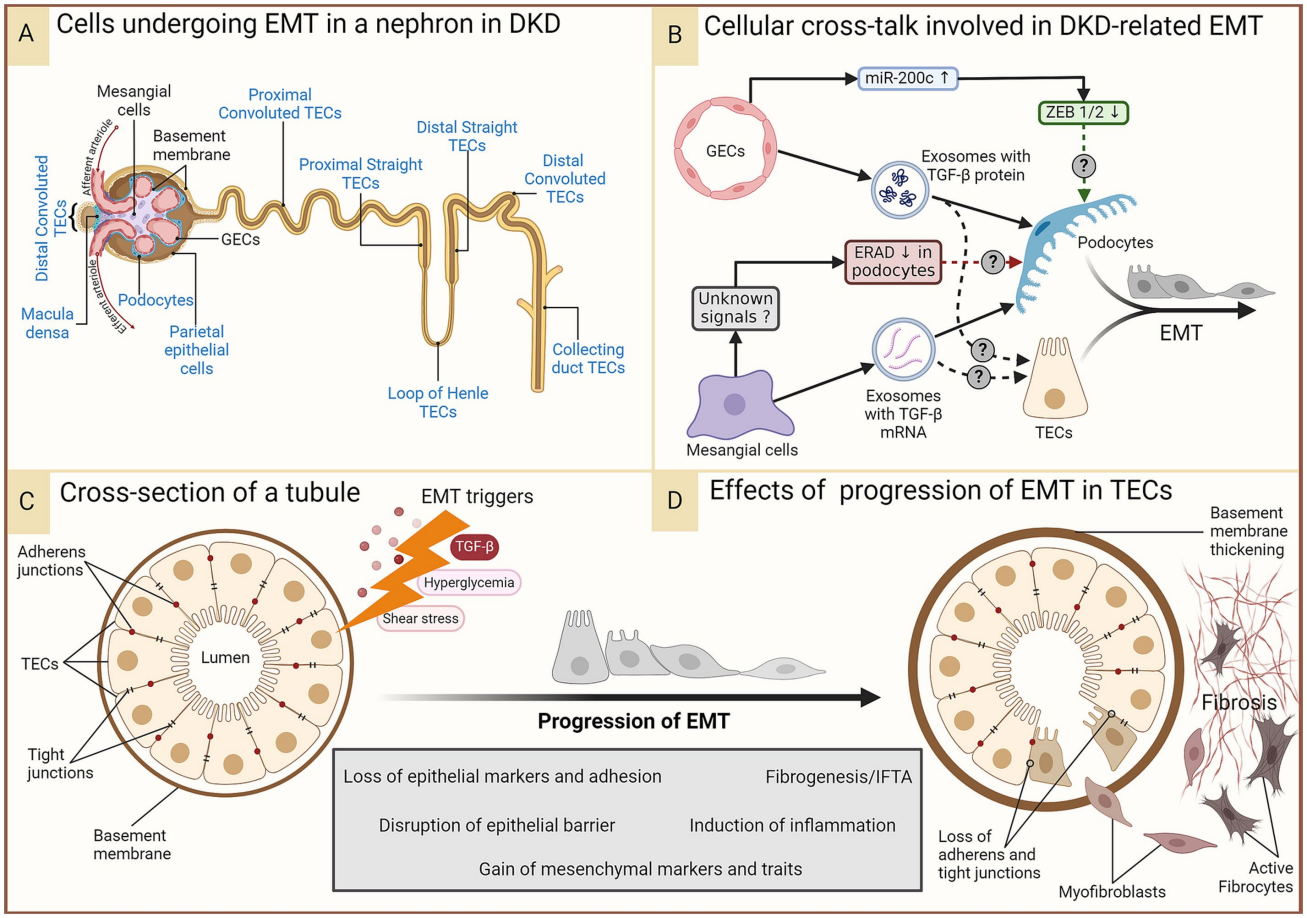
EMT in DKD. In the prolonged diabetic and micro-inflamed milieu, a steady upregulation of EMT-promoting genes induces renal epithelial cells to undergo EMT at least partially, where they lose epithelial characteristics, like cell polarity and cell–cell adhesion, and gain mesenchymal properties. **(A)** Epithelial cells in different parts of the nephron that (potentially) undergo EMT during DKD are indicated in blue. Some of these (podocytes, proximal TECs, and distal TECs) have more evidence-based support, while others have not had as much research attention. **(B)** A summary of renal intercellular cross-talk that influences EMT in the context of DKD. **(C,D)** A schematic representation of the cross-section of a tubule disrupted by constituent cells undergoing EMT induced by the aforementioned triggers, leading to loss of the epithelial barrier function and a growing pool of fibroblasts, their activation, and ultimately, fibrosis. Figure 31.3 of Comprehensive Clinical Nephrology (ISBN: 9780323825924) is acknowledged as an inspiration for the lower panel. DKD, diabetic kidney disease; EMT, epithelial-to-mesenchymal transition; ERAD, endoplasmic reticulum-associated degradation; GECs, glomerular endothelial cells; IFTA, interstitial fibrosis and tubular atrophy; miR-200c, microRNA 200c; TGF-β, transforming growth factor-beta; TECs, tubular epithelial cells; ZEB, zinc finger E-box binding homeobox.

An intricate interplay of intercellular communication within renal cells plays a pivotal role in DKD pathophysiology, including in the epithelial-to-mesenchymal transition (EMT) of renal epithelial cells. Even under normal conditions, communication between podocytes, glomerular endothelial cells (GECs), mesangial cells (MCs), and tubular epithelial cells (TECs) is vital for maintaining the renal architecture. The prolonged diabetic milieu changes intercellular communication, leading to glomerulosclerosis, proteinuria, and eventually renal fibrosis via EMT, amongst other cellular changes. EMT within the glomerulus is characterized by podocytes losing epithelial markers and gaining mesenchymal traits, resulting in a compromised glomerular filtration barrier and fibrogenesis. In the extra-glomerular compartment, EMT of proximal TECs (PTECs), distal TECs, and collecting duct epithelial cells contribute to DKD progression (Figure 3). Recent studies have revealed the signaling molecules, such as coding and non-coding RNAs and transforming growth factor-β (TGF-β), that mediate the cross-talk driving EMT in DKD. These complex interactions involve direct cell-to-cell contact, autocrine, and paracrine mechanisms, such as the secretion of exosomes carrying pro-fibrotic signals to neighboring cells.

TGF-β is a multifunctional cytokine that regulates many cellular functions, including cell growth, differentiation, and apoptosis. TGF-β1, the most widely expressed isoform, drives fibrosis in almost all forms of chronic kidney disease (CKD) by activating both canonical (Smad-based) and non-canonical (non-Smad-based) signaling pathways (Figure 3B) (67). In addition to triggering pre-existing myofibroblast activation, TGF-β1 also expands the myofibroblast pool by inducing EMT of renal epithelial cells and as well as an endothelial-to-mesenchymal transition (EndoMT) of glomerular endothelial cells (16, 67). Wu et al. grew GECs under high glucose stress and showed that these cells produced more exosomes than GECs grown under normal glucose levels; the exosomes were also enriched in TGF-β mRNA. Podocytes internalized these exosomes, triggering EMT in them as indicated by the simultaneous loss of epithelial markers (nephrin, ZO-1, WT-1) and gain of mesenchymal markers (α-SMA, desmin, FSP-1), hallmarks of the podocyte EMT (Figure 3B). Canonical Wnt/β-catenin signaling was shown to be activated by the exosomes (34). Thus, paracrine signaling from GECs in the diabetic milieu might trigger podocyte EMT, leading to more myofibroblasts and furthering fibrosis. While not EMT per se, exosomes secreted by high glucose-treated GECs enriched in TGF-β1 mRNA and circRNAs also trigger MC activation and myofibroblast transdifferentiation, aiding fibrosis (18, 37). High-glucose-induced MCs secrete exosomes enriched in the TGF-β1 protein, and podocytes cultured with these exosomes showed several signs of injury, including a reduction in the expression of nephrin and WT-1, suggesting EMT; however, mesenchymal markers were not tested in the study (29).

TGF-β stressed GECs overexpress miR-200c, which decreases VEGF-A expression and secretion in cultured human podocytes, impairing glomerular homeostasis (68). ZEB1 and ZEB2 are known targets of miR-200c and essential transcription factors playing a role in EMT. Moreover, ZEB1 was shown to be downregulated in podocytes by miR200c overexpression in GECs (68), suggesting cross-talk from GECs protecting podocytes from EMT.

Podocytes exposed to the supernatant of MCs cultured in high glucose conditions show a suppression of the endoplasmic reticulum (ER)-associated degradation (ERAD) (28). Membrane-bound or secretory proteins pass through the ER, where their fidelity is monitored via the ER quality control machinery. ERAD is a complex, conserved process where unfolded/misfolded proteins are recognized, polyubiquitinated, translocated to the cytoplasm, and degraded by the 26S proteasome. Suppression of ERAD causes accumulation of misfolded proteins, leading to ER stress and activation of the unfolded protein response (UPR) to recover protein homeostasis. ER stress induces EMT in human lens retinal epithelial cells (69). UPR signaling is exploited in cancer to promote EMT (70). Whether the ERAD suppression induced by communication from activated MCs ultimately leads to EMT of the podocytes remains a likely but unconfirmed scenario in DKD.

EMT of PTECs is an important contributor to DKD pathogenesis. The glomerular barrier is impermeable to proteins or immune complexes in healthy individuals. Glomerular filtration barrier damage leading to albuminuria is an early event in DKD, even utilized for its diagnosis. It is, therefore, conceivable that the PTEC lumen is exposed to all EMT-promoting and-repressing signals that podocytes are exposed to from the other glomerular cells. Tubular compartments of kidneys of a diabetic animal model of DKD show increased expression of the TGF-β1 type II receptor and the activation of the Smad signaling pathway (71), suggesting that GECs and MCs under hyperglycemic stress can induce EMT in PTECs in addition to podocytes via TGF-β. With increasing kidney injury, excess Angiotensin II and TGF-β induce EMT in PTECs (72).

Understanding the dynamics of the intra-renal intercellular cross-talk is crucial for developing therapies that can target the maladaptive communication that leads to EMT and halt the progression of DKD. Technological advances enable researchers to dissect these cellular dialogues with greater precision, offering hope for new interventions to preserve renal function and improve outcomes for patients with diabetes.

### Renal intercellular cross-talk in EndoMT in DKD

**Figure 4:**
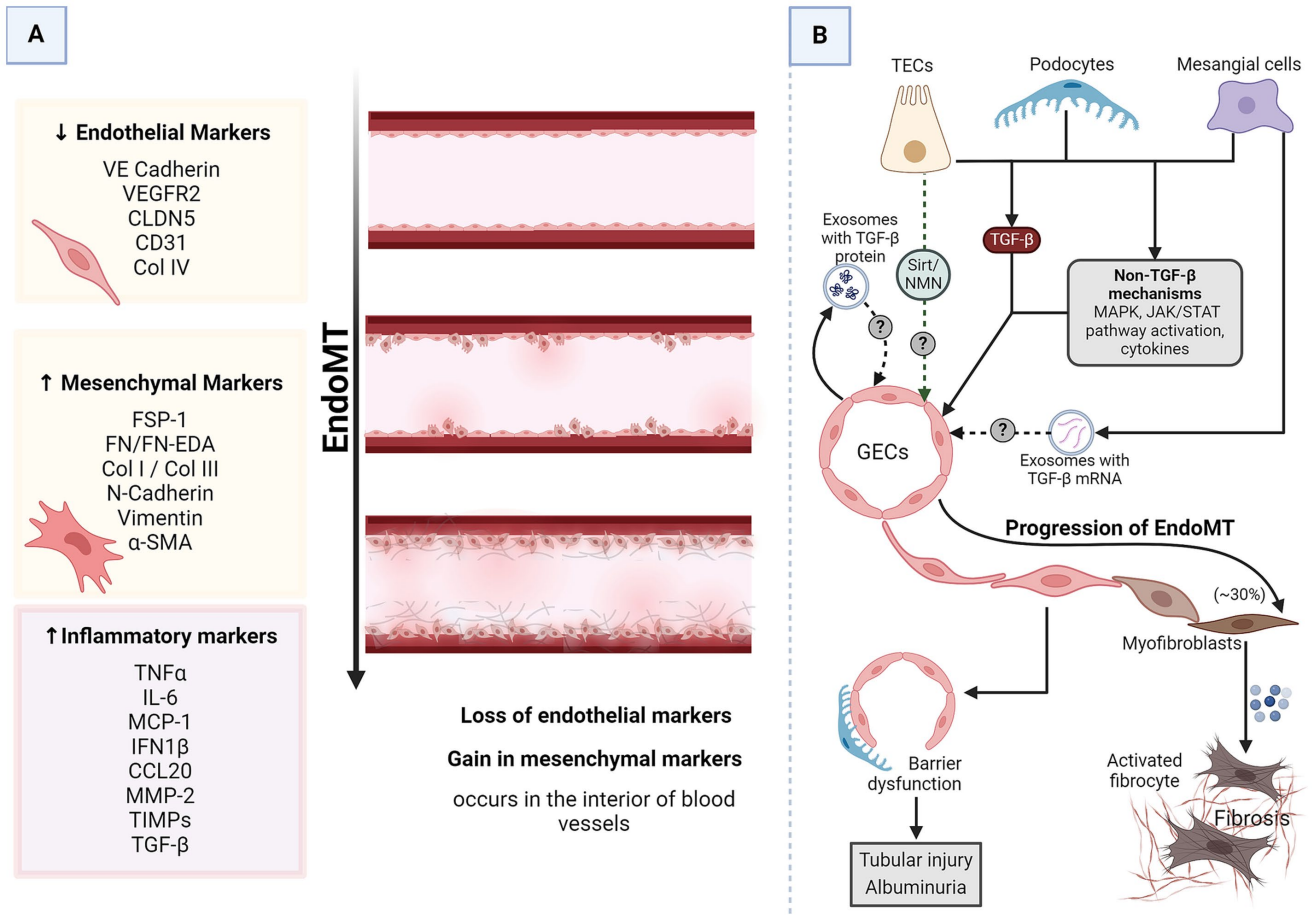
EndoMT in DKD. **(A)** A schematic representation of generalized EndoMT. **(B)** A brief summary of renal intercellular cross-talk that influences EndoMT in the context of DKD. All major renal cell types release signaling molecules capable of influencing EndoMT of GECs, which contributes to the progression of DKD. In the prolonged diabetic and micro-inflamed milieu, a steady dysregulation of EndoMT-related genes induces GECs to undergo EndoMT, where they lose endothelial characteristics and gain mesenchymal properties, leading to increased vascular permeability, increased ECM production, and more myofibroblasts aiding proteinuria, sclerosis, and fibrosis. α-SMA, alpha-smooth muscle actin; CCL20, CC chemokine ligand 20; CD31, cluster of differentiation 31; CLDN5, claudin 5; Col, collagen; DKD, diabetic kidney disease; EDA, extra domain A; EndoMT, endothelial-to-mesenchymal transition; FN, fibronectin; FSP-1, fibroblast-specific protein 1; GECs, glomerular endothelial cells; IL-6, interleukin 6; IFN1β, interferon 1 beta; JAK/STAT, Janus kinase/signal transducer and activator of transcription; MAPK, mitogen-activated protein kinase; MCP-1, monocyte chemoattractant protein 1; MMP-2, matrix metalloproteinase 2; N-cadherin, neural-cadherin; NMN, nicotinamide mononucleotide; Sirt, Sirtuin; TGF-β, transforming growth factor-beta; TECs, tubular epithelial cells; TIMPs, tissue inhibitors of metalloproteinases; TNFα, tumor necrosis factor-alpha; VE-cadherin, vascular endothelial-cadherin; VEGFR2, vascular endothelial growth factor receptor 2.

Endothelial-to-mesenchymal transition (EndoMT) also plays a pivotal role in the pathophysiology of DKD. EndoMT is a process where endothelial cells lose endothelial traits and acquire mesenchymal and fibroblast-like properties (Figure 4A), contributing to the fibrosis observed in DKD (16). GECs maintain the glomerular filtration barrier along with podocytes. EndoMT of GECs induces EMT of the adjacent podocytes, resulting in barrier dysfunction (34). The increased barrier porosity allows the passage of proteins and signaling molecules through the barrier, resulting in tubular cell injury and albuminuria. Additionally, EndoMT of GECs results in an increased pool of myofibroblasts with up to 30% of renal interstitial myofibroblasts in a DKD animal model reported to be of endothelial origin (16, 73). All these processes culminate in progressive fibrosis. This pathogenic cellular transformation of GECs is influenced by the dynamic cross-talk amongst various cell types within the renal corpuscle, including podocytes, MCs, and PTECs. Researchers are beginning to discover the crucial players of this cross-talk (Figure 4B).

TGF-β is the most common driver of most cases of EndoMT, including EndoMT in DKD (16). GECs growing in high glucose conditions undergo EndoMT as evidenced by a decrease in endothelial markers (CD31 and VE-cadherin) while acquiring mesenchymal markers (α-SMA and FSP) (34). As mentioned before, these cells also produce exosomes enriched in TGF-β mRNA. The EndoMT triggered in high-glucose-stressed GECs could result from autocrine signaling via the TGF-β from the released exosomes. MCs under hyperglycemic conditions secrete TGF-β1-enriched exosomes capable of inducing EMT in podocytes (29). Considering the proximity of MCs, podocytes, and GECs, it is plausible that these exosomes can induce EndoMT of GECs via TGF-β1-dependent mechanisms. Podocytes overexpress TGF-β1 in response to excess intraglomerular passage of proteins (74). Tubular compartments of the kidney of diabetic animals show increased expression of TGF-β1 (71). Thus, injured podocytes, activated MCs, and TECs are possible inducers of GEC EndoMT.

Pathways other than TGF-β also induce and regulate EndoMT (16). For instance, NOD2, a pattern recognition receptor, promotes EndoMT in GECs in response to high glucose by activating mitogen-activated protein kinase (MAPK) signaling (75). STAT5A activation increases ELTD1, which induces EndoMT in GECs (76, 77). MAPK and Janus kinase/signal transducer and activator of transcription (JAK/STAT) signaling are immune signaling pathways affected in DKD (78). Endothelial cells undergoing EndoMT release a lot of immune-modulatory chemokines and cytokines (77). All renal cells express pattern recognition receptors and chemokine and cytokine receptors. Thus, GECs undergoing EndoMT can communicate with the other glomerular cells via immune molecules. Sirtuin1 levels in PTECs are reduced even before albuminuria, and increasing PTEC-Sirtuin1 levels protects against glomerular injury and albuminuria in diabetes by maintaining nicotinamide mononucleotide (NMN) concentrations around glomeruli (Figure 1) (41). NMN protects endothelial cells from the damaging signals of IL1β, TNF-α, and angiotensin II (79), all known triggers of EndoMT (80–82). Moreover, NMN has also been shown to be protective against EMT. Thus, it is quite likely that PTECs might interact with and protect GECs from EndoMT.

Thus, almost all major renal cell types (MCs, GECs, podocytes, and PTECs) exposed to a diabetic milieu release signaling molecules capable of inducing or protecting against EndoMT in the adjacent endothelial cells and EMT in the neighboring epithelial cells. The anatomical proximity of these cells allows for a complex communication network mediated by various signaling pathways and molecules, such as the TGF-β/Smad pathway, JAK/STAT pathway, angiopoietins, growth factors, and cytokines. These interactions can lead to changes in cell behavior, resulting in the pathological changes observed in DKD, such as glomerulosclerosis, proteinuria, and fibrosis.

### Renal intercellular cross-talk in MFT of pericytes in DKD

Pericytes are stromal-derived cells that wrap around capillary walls and connect intimately with the adjacent endothelial cells. They are crucial for angiogenesis, vascular stability, and vessel integrity. Unlike retinal pericytes, a renal pericyte can connect with multiple endothelial cells. Some renal pericytes span from the peritubular capillary to the tubule, with processes touching the tubular basement membrane, giving these cells the capability of communicating directly with both endothelial cells and tubular epithelia. In response to kidney injury, pericytes detach from capillary walls, migrate into the interstitial space, proliferate, and transdifferentiate into myofibroblasts, aiding interstitial scar formation and fibrosis (83). While the sources and their proportion of contribution to scar-forming myofibroblasts is a matter of debate, pericytes, along with perivascular fibroblasts, are generally recognized as the primary source of collagen-producing myofibroblasts in kidney fibrosis (53, 84).

Bidirectional communication between pericytes and endothelial cells is essential for this detachment and migration. Blocking either PDGF receptor-β signaling in pericytes or VEGF receptor 2 signaling in endothelial cells attenuates both fibrosis and capillary loss during progressive kidney injury in mice. Blockade of either receptor-mediated signaling pathway prevents pericyte detachment and proliferation (85, 86). In addition to detachment and proliferation, activated PDGF receptor signaling in pericytes also initiates their transdifferentiation into α-SMA and collagen 1-expressing myofibroblasts. Following injury, PGDF expression increases in injured tubules, endothelium, and macrophages in murine models of obstructive and post-ischemic kidney fibrosis. Inhibiting the PDGF-PDGF receptor signaling decreases pericyte differentiation and, therefore, fibrosis (86). As with the other sources, TGF-β signaling also initiates pericyte-to-myofibroblast transition (PMT). Both TGF-β and PDGF signaling are regulated by core fucosylation in initiating pericyte transition (87). Activated PI3K-Akt–mTOR pathway mediates pericyte-to-myofibroblast transition (PMT) by enhancing glycolysis. While inhibiting this pathway reduced PMT of TGF-β-treated pericytes, overexpression of hexokinase II rescued PMT (88). Wnt1 and Wnt4 from various renal sources (proliferating, medullary, and interstitial myofibroblasts, pericytes, and tubular epithelial cells) are also capable of driving myofibroblast transdifferentiation of pericytes (64, 65).

Following pericyte detachment, microvascular endothelial cells are devoid of the protective signaling from pericytes that is essential for microvascular stability. These endothelial cells become prone to injury and dysfunction. Peritubular capillaries are destabilized, leading to microvascular rarefaction (83). Thus, pericytes play a dual role in aiding fibrosis in DKD: (i) pericyte detachment from capillaries destabilizes the microvascular integrity, leading to vascular regression, which results in hypoxic tissues, furthering tissue damage and (ii) detached pericytes proliferate and transdifferentiate into scar-forming myofibroblasts in the renal interstitium directly driving fibrosis.

### Renal intercellular cross-talk in MFT of macrophages in DKD

Macrophage accumulation correlates well with renal fibrosis progression. In addition to their pro-inflammatory role, macrophages can drive fibrosis directly by contributing to the myofibroblast pool. Circulating monocytes originating in the bone marrow differentiate into macrophages that accumulate in the kidneys. The macrophages can undergo myofibroblast transdifferentiation in the process termed macrophage-to-myofibroblast transition (MMT). Macrophage-derived myofibroblasts contribute to kidney fibrosis, as has been evidenced by the findings of co-expression of macrophage and myofibroblast markers, including CD68 or F4/80 (macrophage) and α-SMA (myofibroblast), in a substantial proportion of fibroblast-like cells both in human and experimental fibrotic kidney disease (89). More recently, experiments mapping cell fate in murine models exhibit myeloid-derived myofibroblasts (52). Thus, MMT is an important producer of myofibroblasts in the fibrotic kidney.

Several factors in the renal environment of DKD trigger MMT in macrophages, including hypoxia, hyperglycemia, hyperlipidemia, and an inflammatory milieu. Like the other cell types undergoing MFT, MMT is also controlled by TGF-β/Smad3 signaling with TGF-β1 as the primary inducer. All cells that overproduce TGF-β in DKD (mesangial cells, glomerular endothelial cells, proximal tubular epithelial cells, and infiltrating immune cells like macrophages) are, therefore, potentially signaling macrophages towards MMT. Other major pathways implicated in MMT are adiponectin/AMPK, JAK/STAT, and Wnt signaling pathways. Serum adiponectin levels are elevated in DKD patients. While adipocytes are the primary producers, endothelial cells, inflammatory cells, and epithelial cells have been reported to produce adiponectin. In a murine obstruction model of tubulointerstitial fibrosis, renal interstitial cells showed elevated adiponectin (90). Adiponectin propels MMT in bone marrow-derived monocytes by activating AMPK (90). In cultured mouse monocytes, IL-4 or IL-13 activated STAT6 and induced MMT, as evidenced by the expression of α-SMA and extracellular matrix proteins (fibronectin and collagen I), while in vivo, STAT6 was seen activated in interstitial cells of the obstructed kidney. JAK3 inhibitors or STAT6-deficiency showed less severe fibrosis (91). Both canonical and non-canonical Wnt signaling is elevated in DKD. Because Wnt ligands are ubiquitously expressed, their exact cellular sources involved in DKD remain undefined, but injured tubular epithelia, fibroblasts, and macrophages are known producers. Macrophage-derived Wnt ligands promote β-catenin activation in the tubular epithelium and MFT (92). Tubule-derived Wnt1 induces myofibroblast activation and proliferation (64). Wnt drives renal MMT via canonical Wnt signaling; β-catenin with T-cell factor (TCF) increases MMT, and diverting β-catenin from TCF to Foxo1 inhibits the fibrotic effect of TGF-β and enhances its anti-inflammatory action (93). In a three-way dialogue between macrophages, fibroblasts, and tubular cells, activated Wnt signaling seemingly promotes MMT, FMT, and EMT, respectively.

Exacerbating the situation further, myofibroblast-derived exosomes enhance MMT and kidney fibrosis (94). Deciphering the contents of these exosomes and the myofibroblast-macrophage communication signals would be the next vital step.

## Renal intercellular cross-talk amongst and between immune cells and innate cells in DKD

We summarized renal intercellular cross-talk guiding MFT of macrophages. Other immune cells and their interactions with each other and with resident renal cells play important roles in driving DKD and have been reviewed by others. A comprehensive review would be beyond the scope of this article, but here are a few highlights. Hyperglycemia, hemodynamic abnormalities, and metabolic derangements cause renal cellular injury, which initiates an inflammatory response that requires complex coordinated communication amongst various immune cells and the injured tissues. Local immune cells, fibroblasts, and pericytes get activated and produce a large variety of pro-inflammatory cytokines and chemokines, which results in the infiltration of circulating immune cells near the sites of injury for wound healing. These infiltrated activated immune cells also produce more pro-inflammatory cytokines and chemokines, which further increases inflammation and immune cell infiltration. Prolonged unresolved inflammation and myofibroblast accumulation drive fibrosis, with ECM proteins accumulating in the renal parenchyma, furthering tissue disruption, renal dysfunction, and organ failure (78).

Fibroblasts produce macrophage attractants CCL2 and CCL7 to recruit macrophages, and macrophages produce stromal cell attractants to recruit fibroblasts to aid wound healing and tissue repair in concert. Myofibroblasts also produce CCL2, suggesting that even after transdifferentiation, activated fibroblasts continue recruiting monocytes/macrophages to fibrotic lesions. Other than recruiting each other, macrophages also activate fibroblasts by secreting the necessary factors (M1 macrophages secrete TNF-α, IL-1β, and IL-6, and M2 macrophages secrete TGF-β, fibroblast growth factor (FGF), galectin-3 and activin A). Activated YAP signaling in fibroblasts enhances colony-stimulating factor 1 (CSF1) expression, which recruits and facilitates the proliferation and polarization of macrophages and dendritic cells (95). Epithelial cells are another important source of CSF1. PTEC-specific CSF1 deletion reduces M2 macrophage numbers and increases tubulointerstitial fibrosis in a murine model of acute kidney injury (96). Injured renal epithelia also produce cytokines and chemokines, like MCP-1, RANTES, and fractalkine, which bind their specific receptors on immune cells like macrophages and dendritic cells, activating inflammatory pathways and promoting their migration to the site of injury (56).

Monocytes, M2 macrophages, and T lymphocytes are seen in significantly higher numbers in DKD samples (97). DKD progression correlates directly with proportions of Th1 and Th17 cells and negatively with Treg (regulatory T lymphocytes) numbers (98). Th1 cells express their signature cytokines, IL2, IFNγ, and TNF-α, and thereby participate in the activation of macrophage-mediated immunity. IL17 family cytokines are characteristic of Th17 cells. Depending on the isoform of IL17, they can be pathogenic or protective. IL-17A receptor is mainly expressed in podocytes and tubular epithelial cells. Mohamed et al. found that low-dose IL-17A treatment suppresses podocyte and tubular injury in murine models of type 1 and type 2 diabetes (99). Treg cells suppress T cells, NK cells, NKT cells, B cells, and dendritic cells and regulate the proportion of pro-vs. anti-inflammatory cytokines, thereby affecting intrarenal intercellular communications (100). Research also indicates mast cell, B lymphocyte, and neutrophil involvement, but further research is required to bring clarity to their role in DKD onset and progression (101).

## Cellular cross-talk in the kidney—limitations, preclinical, and clinical correlations

Most research on cellular cross-talk utilizes cells cultured in vitro. As with cell culture studies in general, there are significant limitations when we examine the evidence in animal and human studies. However, cell culture studies have the potential to verify a single hypothesis in a pure and straightforward manner. Cell-to-cell cross-talk, on the other hand, is a unique concept that can be tested in vitro and in vivo studies. While all observations demonstrated in in vitro studies may not be replicated in vivo studies, several published studies have attested to the validity of in vitro data pertaining to cellular cross-talk in the context of DKD.

While most of the work cited in this manuscript pertains to cross-talk between and amongst various cells in the kidney, there are some significant correlations with preclinical and clinical (refer to the next section) as well as histopathology in DKD. The most obvious example of the correlation of cellular cross-talk with renal histopathology in DKD is the role of EMT leading to renal fibrosis mediated by mediators such as Angiotensin II, CTGF, and TGF-β (102).

The cross-talk amongst various renal cells in DKD has been shown to have close correlates in animal and even some human studies. To illustrate this aspect, Tsai et al. demonstrated that miR-92a-1-5p in exosomes derived from proximal tubular epithelial cells treated with high glucose caused mesangial cell injury and induced MFT. This in vitro finding was replicated in vitro murine studies with db/db mice, in which the same authors showed that treatment with an inhibitor of miR-92a-1-5p attenuated kidney damage in these diabetic mice. The authors extrapolated these observations to suggest the potential utility of urinary miR-92a-1-5p assay as a biomarker of DKD in humans (44).

A stronger correlation between the renal cellular talk discussed here and in vitro evidence in DKD was described by Qi et al. in diabetic mice (103). Examining and comparing the transcriptome profile of glomeruli between DKD-resistant C57BL/6 J and DKD-susceptible DBA/2 J (D2). The authors noted significant differences in the degree of mitochondrial dysfunction in the early course of the disease. Increased mitochondrial oxidative stress in mice with DKD was associated with greater levels of circulating Endothelin 1 and enhanced expression of Endothelin receptor type A in the glomerular endothelial cells. The endothelin-mediated mitochondrial dysfunction in the glomerular endothelial cells adversely affects the cross-talk with podocytes, leading to podocyte damage, dysfunction, and depletion. The endothelial-podocyte cross-talk results in albuminuria and glomerulosclerosis.

## Cellular cross-talk in the kidney and its implications in the therapy of DKD

The ultimate test of validity and utility of cellular cross-talk in renal structures is the applications in therapy. One of the most recent pharmacological agents with proven benefits in DKD is the SGLT-2 blockers, which, although introduced initially as drugs to lower hyperglycemia in diabetes, were later demonstrated to decrease the progression of DKD and even heart disease. As already alluded to in the earlier sections, the renoprotective effects of SGLT-2 blockers, particularly anti-proteinuric effects and preservation of GFR, involve significant cross-talk between PTECs and glomerular cells, particularly podocytes. The specifics of such cross-talk in the case of SGLT2 blockers were already discussed in earlier sections.

Inhibitors of RAAS, such as angiotensin-converting enzyme (ACE) inhibitors and angiotensin receptor blockers (ARBs), initially developed 3–4 decades ago to treat hypertension, proved to be extremely effective in preventing the onset and progression of DKD, congestive heart failure, and even coronary artery disease. Although the primary site of action of angiotensin II, the final effect of RAAS was originally presumed on endothelial cells, the currently known actions of angiotensin are much more pleiotropic, suggesting the possibility of significant cell-to-cell cross-talk within the kidney and cardiovascular system. Experimental data suggests that such cross-talk may involve interaction between RAAS and the kallikrein-kinin system, Wnt/β-catenin signaling, and sodium-potassium pump (104).

Another therapeutic class of drugs approved for the treatment of DKD is glucagon-like peptide-1 (GLP-1) receptor agonists, which were also initially developed for the treatment of type II diabetes. Recent clinical trials such as REWIND, SUSTAIN, and FLOW studies have established that these drugs slow down the progression of DKD, although the exact underlying mechanisms remain unclear. The mechanisms of renal protection are elusive since the primary effects of these drugs are on extrarenal sites. GLP-1 receptors have been demonstrated in the smooth muscle cells of preglomerular vasculature. Furthermore, GLP-1 has been shown to inhibit angiotensin II and promote natriuresis. It is therefore suggested that such effects may involve cross-talk between organs and various cells. Studies also have suggested a gut-kidney cross-talk connecting the gut sodium sensing and natriuresis mediated by GLP-1 (105).

Antagonists of mineralocorticoid receptors have also been shown to be renoprotective recently in DKD, although their cardioprotective effects were established a long time ago. The aldosterone receptors in the kidney are in the distal nephron, while their effects in slowing the progression of DKD conceptually should involve cross-talk with glomerular and other renal cells, including those in the interstitium. In experimental animal studies involving transgenic mice, cross-talk between small GTPase Rac1 and mineralocorticoid receptors has been demonstrated that could lead to podocyte injury (106).

Understanding the mechanisms of cellular cross-talk in DKD is crucial for elucidating DKD pathogenesis and identifying potential therapeutic targets. Interventions aimed at modulating these cellular interactions could halt or reverse the progression of DKD. For example, targeting the intercellular cross-talk that mediates MFT could provide a novel approach to reducing renal fibrosis. Additionally, therapies designed to preserve the function of the glomerular filtration barrier by stabilizing the cross-talk amongst renal cells may prove beneficial in managing DKD. Therefore, ongoing research in this area is essential for developing a more comprehensive understanding of DKD and creating innovative treatment strategies that address the complex cellular interactions at play. As our knowledge of these cellular dynamics continues to expand, so does the potential for significant advancements in the care and prognosis of individuals with DKD.

## Conclusion

Diabetes mellitus is turning into a global epidemic, and chronic kidney disease develops in almost half of such patients. There is no optimal therapy to halt the development or progression of DKD since the development of novel therapeutic strategies is hampered by significant gaps in our understanding of the pathogenesis of this disorder. Recent investigations strongly suggest concerted communication cascades constituting continuous cross-talk amongst various cells in the kidney to converge into established pathogenic pathways. Furthermore, it has become increasingly clear that the cell-to-cell transitions, such as EMT and EndoMT, are important steps in the pathogenesis of DKD that lead to final scarring of the kidneys. A concise review of the specifics and role of major cell-to-cell cross-talk in the early and late stages of DKD, as well as the EMT and EndoMT, are presented in this article. The expansion of the existing knowledge of EMT and cellular cross-talk in DKD will further enlighten us on the complex pathogenic mechanisms and ensure more effective therapeutic strategies to slow down DKD.

## Glossary

8-OHdG: 8-hydroxy-2'-deoxyguanosine
α-SMA: alpha-smooth muscle actin
ACE: angiotensin-converting enzyme
AGE: advanced glycosylation end products
AMPK: adenosine monophosphate (AMP)-activated protein kinase
ARBs: angiotensin receptor blockers
BAMBI: bone morphogenetic protein and activin membrane-bound inhibitor
BMPs: bone morphogenetic proteins
cGMP: cyclic guanosine monophosphate
circRNA: circular RNA
CKD: chronic kidney disease
cNOS: constitutive nitric oxide synthase
CSF1: colony-stimulating factor 1
CTGF: connective tissue growth factor
DKD: diabetic kidney disease
DM: diabetes mellitus
ECM: extracellular matrix
EGR 1-3: early growth response 1-3
EMT: epithelial-mesenchymal transition
EndoMT: endothelial-to-mesenchymal transition
eNOS: endothelial nitric oxide synthase
ERAD: endoplasmic reticulum-associated degradation
ERS: endoplasmic reticulum stress
ET-1: endothelin 1
FGF: fibroblast growth factor
FMT: fibroblast-to-myofibroblast transition
FSP-1: fibroblast-specific protein 1
GBM: glomerular basement membrane
GECs: glomerular endothelial cells
GFR: glomerular filtration rate
GLP-1: glucagon-like peptide-1
GR: glucocorticoid receptor
HG: high glucose
IFTA: interstitial fibrosis and tubular atrophy (used 3X, defined every time)
IL: interleukin
iNOS: inducible nitric oxide synthase
JAK/STAT: Janus kinase/signal transducer and activator of transcription
MAPK: mitogen-activated protein kinase
MCP1: monocyte chemoattractant protein 1
MCs: mesangial cells
MFT: myofibroblast transdifferentiation
miRNA: microRNA
MMP: matrix metalloproteinases
MMT: macrophage-to-myofibroblast transition
mTORC: mechanistic target of rapamycin complex
NADPH: reduced nicotinamide adenine dinucleotide phosphate
NF-κB: nuclear factor kappa-light-chain-enhancer of activated B cells
NK cells: natural killer cells
NKT cells: natural killer T cells
NMN: nicotinamide mononucleotide
NO: nitric oxide
NOS: nitric oxide synthase
NRP1/2: neuropilin 1/2
PCs: podocytes
PDGF: platelet-derived growth factor
PDGFR: platelet-derived growth factor receptor
PI3K: phosphatidylinositol 3-kinase
PMT: pericyte-to-myofibroblast transition
PTECs: proximal tubular epithelial cells
RAAS: renin-angiotensin-aldosterone system
RANTES: regulated upon activation, normal T cell expressed and secreted
ROCK: Rho kinase
ROS: reactive oxygen species
SEMA3C: semaphorin 3C
SGLT-2: sodium-glucose cotransporter 2
STZ: streptozotocin
TCF: T-cell factor
TECs: tubular epithelial cells
TEMPO: 2,2,6,6-tetramethylpiperidine-1-oxyl
TGF-β: transforming growth factor beta
TIMP: tissue inhibitors of metalloproteinases
TNF-α: tumor necrosis factor-alpha
UPR: unfolded protein response
VEGF: vascular endothelial growth factor
VEGFR: vascular endothelial growth factor receptor
WT-1: Wilms tumor 1
ZO-1: zonula occludens-1
